# Shockwave Therapy Efficiently Cures Multispecies Chronic Periodontitis in a Humanized Rat Model

**DOI:** 10.3389/fbioe.2019.00382

**Published:** 2019-12-13

**Authors:** Akshay Datey, C. S. Adeeb Thaha, Sudhir R. Patil, Jagadeesh Gopalan, Dipshikha Chakravortty

**Affiliations:** ^1^Department of Microbiology and Cell Biology, Indian Institute of Science, Bangalore, India; ^2^Department of Aerospace Engineering, Indian Institute of Science, Bangalore, India; ^3^Centre for Biosystems Science and Engineering, Indian Institute of Science, Bangalore, India; ^4^Department of Periodontics, K.L.E. Society's Institute of Dental Sciences, Bangalore, India

**Keywords:** shock waves, biofilms, periodontitis, patient sample, humanized rat model

## Abstract

Biofilms are ubiquitous in nature and are invariably associated with health and diseases of all living beings. Periodontal diseases & dental caries are the most prevalent conditions in which biofilm has established as a primary causative factor. Managing poly-microbial biofilm is the mainstay of periodontal therapy. Plethora of antimicrobials have been used till date to combat biofilm, but the emergence of antibiotic tolerance and resistance in biofilms is a major cause of concern. Apart from use of antimicrobials, various anti-biofilm strategies have evolved which include the use of mechanical, and chemical means to disrupt biofilms. However, none of these approaches have led to desired or optimal biofilm control and hence search for novel approach continues. Shockwaves are used in medical practice for various therapeutic purposes and in local drug delivery, gene therapy, wound healing & regeneration. With this background, a study was designed with an attempt to explore the possibility of using the shockwave for their effect on multispecies oral biofilm development from subgingival plaque samples obtained from chronic periodontitis patients. Plaque samples from 25 patients were used to derive multispecies biofilm which were used to check the efficacy of shockwaves and antibacterial efficacy of four clinically relevant antimicrobials. Biofilms were analyzed by scanning electron microscope; atomic force microscope and their biomass was quantitated by crystal violet staining. Further, a humanized rat model of periodontitis was developed. Patient derived plaque was used to establish periodontitis in healthy rats. The model was validated by performing colony forming unit (CFU) analysis of the infected tissue. The animals were subjected to low intensity shockwaves using a hand-held shockwave generator at the site of infection. Shockwave treatment was done with or without antimicrobial application. The animals were monitored for clearance of infection and for mortality. The results show that shockwave treatment in combination with antimicrobials is significantly effective in clearing a multispecies biofilm. This also brings out the possibility of application of shockwaves in the management of oral biofilms either alone or in combination with established antimicrobial agents. With further research, safety profile validation and clinical trials, shockwaves can be an effective, novel approach in management of biofilm associated periodontal disease.

## Introduction

Human oral cavity is a complex ecological environment which is conducible for the growth and survival of microorganisms. Dental plaque is a dynamic and complex polymicrobial biofilm which consists of more than 700 species of bacteria, adhering to surfaces or interfaces, and are usually embedded in an extracellular matrix (ECM) (Paster et al., 2006). These diverse microbial residents form a highly organized biofilm structures within the community displaying extensive interactions, inducing microbial pathogenesis under certain conditions and carrying out sophisticated physiological functions and have a dynamically complex biological system (Kolenbrander et al., 2002). Hence the number of bacterial species within the plaque is currently undetermined. Dental plaque develops at stagnant and difficult to reach sites between the teeth, the tooth–gingival interface or in the pits and fissures on the occlusal surfaces of the posterior teeth. In periodontally healthy conditions, in the tooth–gingival interface the supragingival plaque is dominated by gram-positive bacteria, with high proportions of streptococcus species. As inflammation develops (gingivitis), this microbiota shifts to a more gram-negative, anaerobic bacterium species. When the periodontal lesion establishes, gram-negative anaerobic species, especially *Porphyromonas gingivalis, Tannerella forsythia, Actinomyces* spp., and *Treponema denticola* predominate. Dysbiosis of subgingival organisms triggers periodontal diseases and a single periodontal pocket harbors a complex polymicrobial community (Socransky and Haffajee, 2002). Biofilms not only help protect the resident organisms from both immune mechanisms and antimicrobial agents as the subgingival microbiota is very organized but also impart higher virulence potential and antimicrobial resistance hence a challenge in the of control periodontal infections (Kazor et al., 2003). Further the host immune-inflammatory response to bacterial products released from the oral biofilm results in exacerbated inflammation which cause host tissue damage and ultimately rapid bone destruction (Gibbons, 1989). Various antibiofilm strategies have evolved right from mechanical/physical, chemical and biological approaches, Antimicrobials used locally and systemically, phytochemicals (Plant extracts, Honey, tea tree oil) are been used traditionally. New approaches like guiding periodontal pocket recolonization, nanotechnology, and photodynamic therapy are also being employed with variable but promising adjunctive modalities for biofilm management in periodontal therapy (Sadekuzzaman et al., 2015). The physical removal of plaque biofilm by meticulous brushing is established and most effective mode for the maintenance of healthy gums and teeth. Plethora of antimicrobials have been used till date as adjuncts to combat oral biofilms, but antibiotic tolerance and enhanced resistance in biofilm and the high Minimum Inhibitory Concentration (MIC) and Minimum Bactericidal Concentration (MBC) of biofilm cells as demonstrated in experimental studies, are still the areas of concern (Wang et al., 2012). Various *in vitro, ex vivo*, and *in vivo* biofilm models have been investigated to study the complex oral biofilm ecosystem and efficacy of different modalities of its control in periodontal disease management (Darveau et al., 1997).

Shockwaves (SW) are disturbances in aerodynamics traveling at supersonic speeds and are virtually independent of the wave amplitude (Jagadeesh, 2008). Extracorporeal shock wave therapy (ESWT) has been enormously used in medical practice, principally, for the management of urolithiasis, cholelithiasis and in various orthopedic and musculoskeletal disorders (Porfyris et al., 2011; Datey et al., 2016; Loske, 2017). Various studies conducted to evaluate the bactericidal efficacy of shock waves in destruction of bacteria have given controversial results. One such study showed that shock waves at high energy levels have a lethal effect on bacteria (Gerdesmeyer et al., 2005), while other study concluded that the microorganisms continued to persist and cause inflammation in the sites treated with shock wave (Sathishkumar et al., 2008). The recent medical reports of use of shock waves (SW) for various therapeutic purposes have opened new vistas to be explored for dental applications (Datey et al., 2016). Anti-bacterial effects of shock waves have shown promising results in some studies (Gnanadhas et al., 2015), however there is a lack of substantial research and cumulative evidence about this in the available dental literature. With this background the present *in-vitro* study was designed with an aim to assess the effect of shock waves on multispecies oral biofilm and its adjunctive effect on the antibacterial efficacy of four different antimicrobial agents commonly used in periodontal therapy as a novel approach.

## Methods

### Generation of Shock Waves Using Hand-Held Device

**Figure 1:**
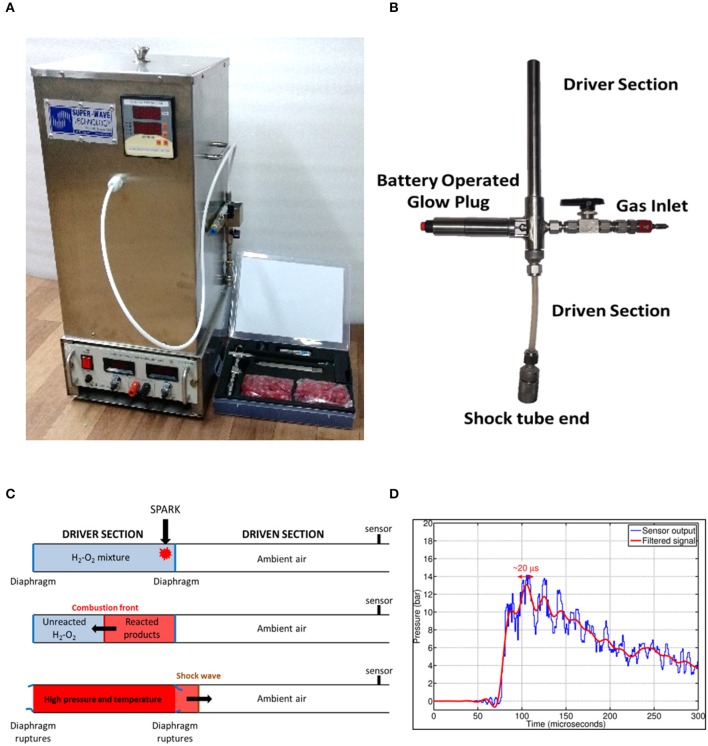
Design and working principle of shockwave-based device for dental applications. **(A)** Oxyhydrogen driven miniature 6 mm shock tube assembly. **(B)** Handheld assembly used for dental application. The device consists of driver and driven sections, battery operated glow plug and a gas inlet. The flexible driven section enables the user to conviniently position shockwave exposure. **(C)** Working principle of the device. **(D)** Typical pressure vs. time profile generated using the device depicting shockwave formation.

An oxyhydrogen detonation-driven miniature shock tube assembly to generate shockwaves of required strength and duration has been reported (Janardhanraj and Jagadeesh, 2016; Datey et al., 2017; Subburaj et al., 2017). A similar experimental setup with slight modifications has been used for the present work (Figures 1A,B). The device comprises of two main components—an oxyhydrogen generator and a miniature shock tube assembly. The oxyhydrogen generator produces the required amount of stoichiometric mixture of hydrogen and oxygen gases through alkaline electrolysis. A miniature shock tube assembly with an internal diameter of 6 mm is used. The oxyhydrogen mixture is filled in the driver section of the shock tube and a battery-operated glow plug is placed close to the diaphragm between the driver and the driven section. This is used to ignite the mixture to produce a detonation front. The high pressure and temperature behind the detonation front causes the instantaneous rupture of the diaphragm between the driver and driven section and produces a strong shockwave in the driven section of the shock tube (Figure 1C). Tracing paper (95 GSM) is used as diaphragm in the shock tube. The sample is exposed to shock waves produced at the end of this setup. For all the experiments in this study, 2.5 bar fill pressure was maintained for shock wave generation. The peak amplitude and the steady time of the shockwave generated were 14 bar and 20 μs respectively (Figure 1D).

### Patient Identification

Salivary and plaque samples were collected from 25 patients of both genders diagnosed with chronic periodontitis in the outpatient division of the Department of Periodontics, KLE Dental College, Bangalore. A written informed consent was obtained from each subject recruited for the study. Ethical clearance for collection of salivary and plaque samples as well as for the study was obtained from the Institutional Ethics Committee.

### Selection and Inclusion Criteria

Systemically healthy subjects of both genders in the age group of 20–50 years with the presence of at least 20 teeth in the mouth diagnosed with generalized chronic periodontitis (severe). Sampling sites should include bleeding on probing, a pocket depth ≥7 mm, and/or attachment loss ≥ 4 mm.

### Exclusion Criteria

- Subjects with systemic diseases like diabetes, hypertension, heart disease and rheumatoid arthritis that could alter the course of periodontal disease.
- Subjects having taken antibiotic/anti-inflammatory/immunosuppressive medication in the previous 6 months.
- Subjects who have undergone periodontal therapy in the previous 6 months of the study.
- Pregnant women and lactating mothers.
- Chronic smokers.

### Saliva Collection

Patients were asked to rinse with distilled water thoroughly prior to donating saliva and to spit directly into the saliva collection tube. Five milliliters of unstimulated saliva was collected in a collection tube. Samples were diluted to 1:10 with sterile Phosphate Buffered Saline (PBS) and were filter sterilized.

### Collection of Subgingival Plaque Samples

Subgingival plaque samples were collected from the same patients who donated saliva using an area specific Gracey-curette (Hu-Friedy, Chicago, USA). Criteria used for selection of the patients included bleeding on probing, a pocket depth ≥7 mm, and attachment loss ≥4 mm.3 sites of deepest periodontal pockets were identified. Selected sites and the adjacent teeth were isolated with cotton roll; supragingival plaque was carefully removed with a sterile scaler to prevent any possible contamination of the subgingival plaque samples with saliva or supragingival plaque. The curette was inserted gently as deep as possible into the pocket and as soon as the curette met the tissue resistance at the apical part of the pocket, subgingival sampling was obtained with one single vertical stroke. The plaque sample was immediately transferred to 1.5 ml aliquot of Reduced Transport Fluid (RTF) and stored in an ice bucket at 4°C until transported to microbiological lab.

### Preparation of Natural Hydroxyapatite (HAP) Discs

**Figure 2:**
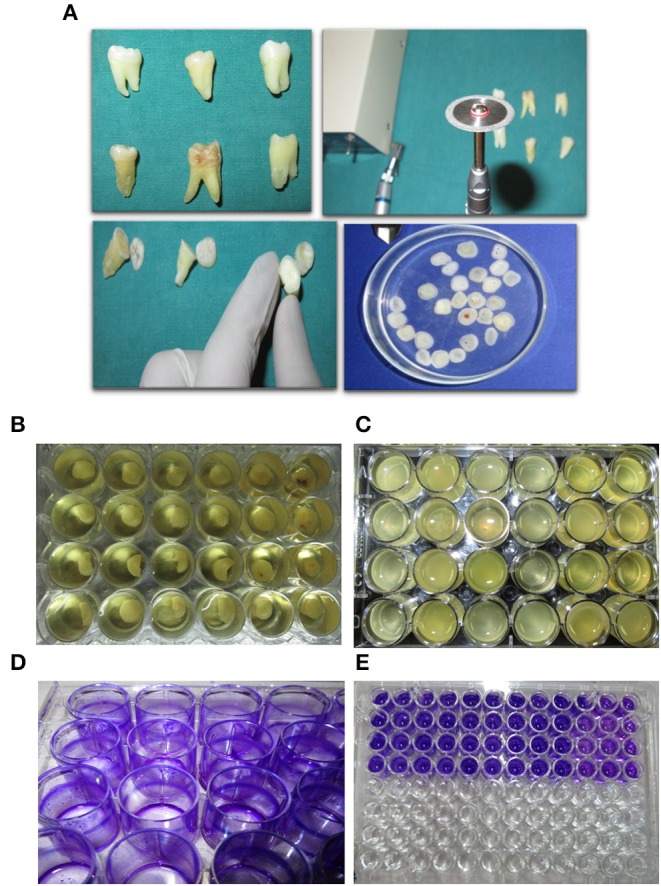
Experimental approach and outline. **(A)** Dentine disc preperation from excised tooth using a diamond cutter. **(B)** Initiating a polymicrobial biofilm in a 24-well plate on dentine discs. **(C)** Mature 7 day-old biofilm formed on dentine discs. **(D,E)** Biofilm biomas qunatification using crystal violet staining.

Dentin slices, ~2 mm thick were cut from the crown sections of extracted human molar teeth slightly below the cementum-enamel junction (CEJ), using a double-sided diamond disk operated on micromotor with water cooled mechanism (Aseptico -M4B-27755, USA) and a Straight handpiece (Uniq—Kavo, GERMANY). A total of 50 extracted teeth were used in this study. Dentin discs were checked for integrity and absence of lesions before including them in the experiments. Discs not found suitable were discarded. Dentin slices were then polished using a 200 and 1,000 grit wet paper to create an even and uniform surface. Dentin/HAP discs were then washed with deionized water to remove the polishing abrasive and finally stored in phosphate buffered saline (PBS) (Figures 2A,B).

### Experimental Design

To assess the effect of various antimicrobial agents used in the control of multispecies oral biofilms developed from subjects with chronic periodontitis. Further the biofilms were subjected to the application of shockwaves and studied for the effect on the biofilm when applied alone and when applied in conjunction with the antimicrobials tested in the study. All the procedures were performed on a 7-day old *ex vivo* biofilm. Each patient sample was divided according to the following scheme of classification for the experiment.

**Control group**
  **Group I**: Control biofilm

**Study Group:**
  **Group II: Biofilm exposed to only Shock Waves (SW)**
  **Group III: Biofilm exposed only to Antimicrobials (AMA)**
    AM 1: Biofilm exposed to Amoxicillin: Metronidazole (AMX-MTZ)
    AM 2: Biofilm exposed to Tetracycline (TET)
    AM 3: Biofilm exposed to 0.25% Sodium hypochlorite (NaOCl)
    AM 4: Biofilm exposed to 0.12% Chlorhexidine (CHX)
  **Group IV: Biofilms exposed to Shock Waves followed by Antimicrobials**
    SW AM 1: Biofilm exposed to SW—AMX-MTZ
    SW AM 2: Biofilm exposed to SW—TET
    SW AM 3: Biofilm exposed to SW—NaOCl
    SW AM 4: Biofilm exposed to SW—CHX.

### Biofilm Development

Sterile HAP disks/coverslips were coated with 10% filter sterilized saliva overnight at room temperature placed in a 24 well tissue culture plate. Three milliliters of Brain Heart Infusion (BHI) media and 20 μl of vortexed subgingival plaque was added into each well. The disks were then incubated under anaerobic conditions at 37^0^C using the Anaerogas Pack (Himedia) for a period of 7 days (Figures 2B,C). Control group biofilm grown disks were removed on the 7th Day from the media and processed for imaging and quantification.

### Evaluation of the Effect Antimicrobials on Developed Biofilms

Four antimicrobial formulations were tested against the developed biofilms. The 7-day HAP discs having the biofilms were transferred to a new 24 well plate with the addition of fresh BHI media. Biofilms of the first column of each plate were removed to establish the baseline values for biofilm quantification and imaging. Antimicrobials namely Amoxicillin-metronidazole (AMX-MTZ), tetracycline (TET), 0.12% Chlorhexidine (CHX) and 0.25% Sodium hypochlorite (NaOCl) were added into each row and left undisturbed for 24 hrs. The biofilms were then imaged under SEM/AFM and were quantified.

### Quantification of Biofilm: Crystal Violet (CV) Staining of Biofilms

The HAP disks/coverslips were washed with phosphate buffered saline (PBS) three times to remove the planktonic cells. The coverslips were dried and stained with 1% (w/v) crystal violet for 20 min at room temperature. After three PBS washes, the bound crystal violet was solubilized with 100 μl of 70% ethanol. The optical density (OD) was determined at 650nm (SpectraMax 340PC, Molecular Devices) (Figures 2D,E).

### Shock Wave Assisted Disruption of Biofilms

The 24 well plate containing the polymicrobial biofilm grown on dentine disks was covered with parafilm and the end of the shock tube was placed on top. Shockwave treatment was done uniformly for all the samples. The biofilms were further analyzed by CV staining, scanning electron microscopy (SEM) and AFM to validate the disruption caused by shock waves (**Figures 4**, **5**).

### Scanning Electron Microscopy (SEM)

HAP discs with biofilm were fixed with 2.5% (v/v) glutaraldehyde and the samples were dehydrated with increasing concentrations of ethanol for 2 min each. The samples were stored in vacuum until use. Prior to analysis by Field emission SEM (FEI-SIRION, Eindhoven, Netherlands), the samples were subjected to gold sputtering (JEOL JFC 1100E Ion sputtering device).

### Atomic Force Microscopy (AFM)

The biofilm samples were collected using a sterile glass piece and dried in a desiccator and visualized using NX-10 Atomic force microscope, Park Systems under non-contact mode.

#### Extraction of DNA and PCR

Total DNA from the *in-vitro* grown biofilm was isolated using DNeasy Blood & Tissue Kit procured from Qiagen technologies ltd. Manufacturers protocol was followed unless mentioned otherwise. Isolated DNA was quantified using Nanodrop ND2000. Two hundred nanograms of DNA was taken as a template for PCR. Primers specific to the target bacteria were used for amplification. The PCR products were analyzed by agarose gel electrophoresis.

### List of PCR Primers

**Table:** 

**Microorganism**	**Primer**
*Aggregatibacter actinomycetemcomitans*	F: AAACCCATCTCTGAGTTCTTCTTC R: ATGCCAACTTGACGTTAAAT
*Actinomyces neslundii*	F: CGCCCTTTTTTGGTGTTTTTGG R: CACCCACAAACGAGGCAGGCCTG
*Porphyromonas gingivalis*	F: AGGCAGCTTGCCATACTGC R: CTGTTAGCAACTACCGATGT
*Streptococcus oralis*	F: TCCCGGTCAGCAAACTTCAGCC R: GCAACCTTTGGATTTGCAAC
*Veillonella parvula*	F: GTAACAAAGGTGTCGTTTCTCG R: GCACCRTCAAATACAGGTGTAGC
*Fusobacterium nucleatum*	F: CGCAGAAGGTGAAAGTCCTGTAT R: TGGTCCTCACTGATTCACACAGA

### Humanized Rat Model of Periodontitis and Shockwave Treatment

Eight weeks old, male Sprague Dawley rats were used to establish a humanized rat model of periodontitis. Plaque samples from chronic periodontitis patients was collected as described earlier. Five different plaque samples were pooled and concentrated by centrifugation. Two hundred microliters of the concentrated plaque was injected using 26G syringe into the sub-gingival pockets in the mesial direction as depicted in **Figure 5**. The animals were incubated with normal food and water for 21 days. The animals were monitored for disease progression and mortality. Diseased rats were randomly segregated into the following 4 groups (3 animals/group): control, antimicrobials alone, shockwave alone and shockwave plus antimicrobial treatment. Antimicrobials were systemically administered whereas shockwaves were topically applied.

### Statistical Analysis

Graph production, data distribution, and statistical analysis were performed using GraphPad Prism (Version 5; La Jolla, CA, USA) software. Intragroup comparisons were done by One-way Analysis of Variance (ANOVA) test followed by Tukey *post-hoc* test. Student *t*-test was employed to investigate significant differences between the independent sample groups.

## Results

### Evaluation of Biofilm Formation

**Figure 3:**
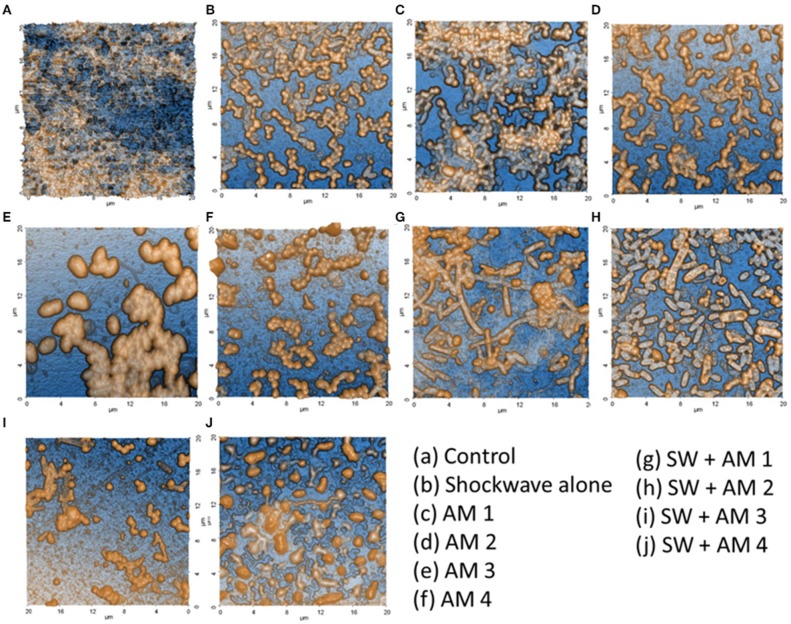
Validation of polymicrobial biofilm formation by atomic force microscopy. Biofilms formed on the dentine discs were analyzed by atomic force microscopy. **(A)** Control fully formed biofilm depicting its polymicrobial nature. **(B)** Effect of shockwave treatment on polymicrobial biofilm. **(C–F)** Effect of antimicrobial agents alone on the biofilm. **(G–J)** Combinatorial effect of shockwaves and antimicrobials on the biofilms grown on dentine discs. The images highlight the disruption of biofilms as well as the reduction of extracellular polymeric substance (EPS) upon various treatments.

**Figure 4:**
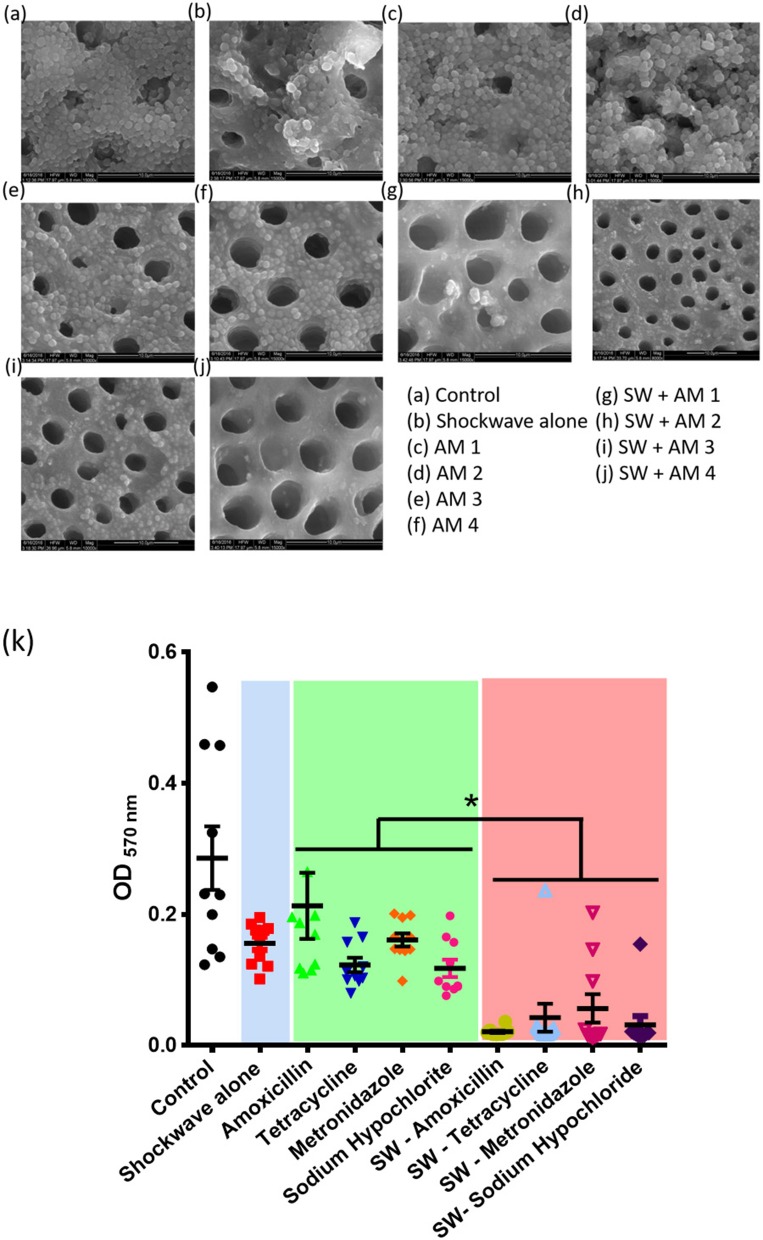
Effect of shockwave therapy on the efficacy of antimicrobials. Scanning electron micrographs of **(a)** Control bilofilm, **(b)** Shockwave treated biofilm, **(c–f)** Biofilms treated with antimicrobials alone and **(g–j)** Biofilms treated with shock waves followed by respective antimicrobial treatment. **(k)** Combined data of 25 patient samples subjected to the above scheme of treatments. Shockwave followed by amoxicillin treatment yields the best biofilm disruption and clearance. *P < 0.05.

The biofilms were grown on saliva coated natural HAP discs in an anaerobic condition for a period of 7 days. The extent of biofilm formation on HAP discs was determined using scanning electron microscopy (SEM) and AFM (Figures 3, 4). The subgingival plaque formed biofilms under the conditions used. The PCR products obtained by species specific primers of the six periodontopathogens were analyzed by agarose gel electrophoresis. Biofilms were grown in the presence of saliva on HAP discs and sterile glass coverslips as determined by SEM and AFM, respectively and the increase in biomass attached to the HAP surface was seen by crystal violet staining for biofilms. When the Control group (Group I) was compared to all the test groups (Group II, III, IV) we observed a statistically significant difference. This indicates that any treatment modality as performed in this study significantly reduces the biofilm biomass when compared to the control biofilm.

### Effect of Antimicrobials Alone on the Polymicrobial Biofilm (Group III)

**Figure 5:**
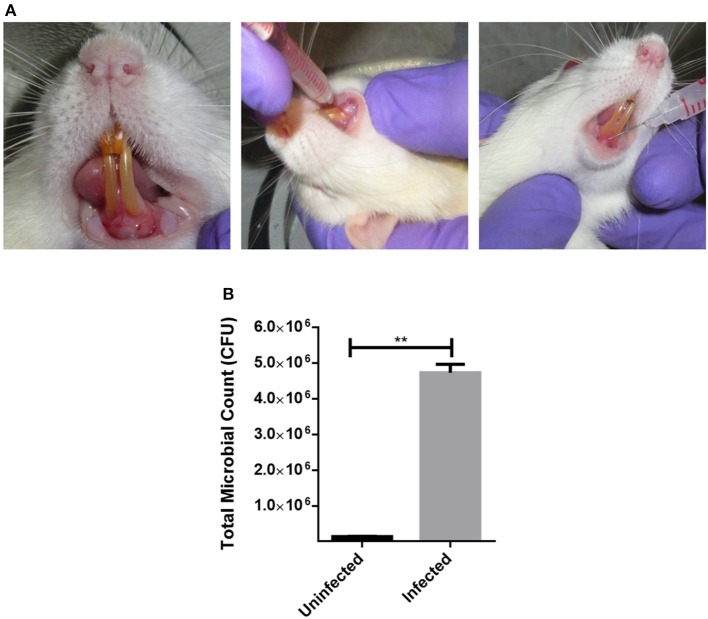
Establishing a humanized model of periodontitis in rats. **(A)** Sprague Dawley rats were injected with pooled plaque samples from 5 chronic periodontitis patients. To establish a humanozed model of periodontitis, they were incubated for 21 days with normal food and water. **(B)** Post-incubation, the total CFU from the site of infection was ennumerated to confirm the disease establishment. **P < 0.005.

The effects of AMX-MTZ, TET, NaOCl, and CHX were independently determined on biofilms grown to the climax stage (7 days old) by exposing climax-grown biofilms to a single antibiotic concentration for a 24-h period and determining the biofilm load by spectrophotometric analysis. SEM and AFM images showed reduction in the biofilm on addition of various antimicrobials (Figures 4, 5). Intragroup comparison among the antimicrobial group (Group III) was performed using the ANOVA test. There was a significant difference (^**^) in the efficacy of TET (0.12 ± 0.32) when compared to AMX—MTZ (0.17 ± 0.52). No significant difference was observed when AMX—MTZ was compared to NaOCl, TET compared to NaOCl and TET compared to CHX demonstrating that all the antimicrobials had significantly reduced the biofilm. A highly statistically significant difference (^***^) was observed when CHX (*p-*value = 0.09 ± 0.009) was compared to AMX – MTZ (*p*-value = 0.17 ± 0.52) and NaOCl (*p*-value = 0.15 ± 0.03) reaffirming the fact that Chlorhexidine is an established and efficient antimicrobial agent in oral biofilm management. However, no significant difference was observed when CHX (*p*-value = 0.09 ± 0.009) was compared with TET (*p*-value = 0.12 ± 0.32), both being equally effective in biofilm reduction (Table 1).

**Table 1:** Results of student *t*-test comparing antimicrobials alone with the Shockwave antimicrobial group.

**Group**	**Mean ± Std. Deviation**	***p*-value**	**Significance**	***t*-value**	**df**
AM 1 v/s SW-AM 1	0.17 ± 0.52 0.03 ± 0.02	*p* < 0.001	^***^	7.305	22
AM 2 v/s SW-AM 2	0.12 ± 0.33 0.05 ± 0.04	*p* < 0.001	^***^	4.808	
AM 3 v/s SW-AM 3	0.15 ± 0.03 0.07 ± 0.06	*p* < 0.001	^***^	4.262	
AM 4 v/s SW-AM 4	0.09 ± 0.009 0.02 ± 0.003	*p* < 0.001	^***^	28.343	

*** *P < 0.0005*.

### Effect of Shockwaves Alone on Biofilms (Group II)

Seven-day old biofilms when subjected to shockwave revealed that the shockwaves had reduced the biofilm load, evaluated by SEM and AFM image and spectrophotometric analysis (Figures 3, 4). It was observed that the dense complex biofilm structure was disrupted into multiple isolated colonies of microorganisms on exposure to SW.

### The Combined Effect of Shockwave Exposure and Antimicrobial Agent's Treatment on the Biofilm (Group IV)

The climax biofilm was first subjected to shockwave and followed by addition of four antimicrobial agents independently. The results showed that, while biofilms were structurally resistant to individual antimicrobials tested, the biofilms became susceptible to antimicrobials after treatment with the shockwaves which had drastically reduced the biofilm content, as observed under AFM & SEM. Interestingly among the shockwave antimicrobial group (Group IV), none of the subgroups compared showed a significant difference when evaluated quantitatively by CV staining. This shows that after shockwave application, biofilms were significantly reduced irrespective of antimicrobials used. Thereby, it can be concluded that shockwave exposure causes physical disruption of the dense biofilm making it susceptible to antimicrobial agents.

### Intergroup Comparison Between Antimicrobial Agent Group (Group III) and Shockwave + Antimicrobial Group (Group IV)

A stringent statistical test (student's *t*-test) was applied to compare between the antimicrobial alone (Group III) to respective antimicrobial following shockwave application (Group IV). Antimicrobials alone had reduced the biofilm load, but the adjunctive use of shockwave had a significantly (*p* < 0.0001) higher effect on the biofilm reduction. Table 2 shows the *p*-value of the study groups in ascending order of which SWAM4 (*p*-value = 0.02) showed the highest significance value followed by SWAM1 (*p*-value = 0.03) and SWAM2 (*p*-value = 0.05). However, the reduction in biofilm biomass observed was in the following order:

SWAM4> SWAM1> SWAM2> SWAM3 > AM4> AM2> SW> AM3> AM1.

This result highlights that although shockwave treatment enhances the efficacy of all the antimicrobials tested, chlorhexidine treatment shows the maximum efficacy enhancement (Figure 4k).

**Table 2:** Results of *post-hoc* (Tukey) test comparing control group with the other test groups.

	**Parameters**	
**Control v/s**	**Mean difference (95% CI)**	***p*-value**
SW	0.109 (0.03–0.18)	<0.0001^***^
AM 1	0.088 (0.01–0.16)	0.001^**^
AM 2	0.136 (0.05–0.21)	<0.0001^***^
AM 3	0.105 (0.02–0.18)	0.002^**^
AM 4	0.166 (0.08–0.24)	<0.0001^***^
SW AM1	0.234 (0.15–0.31)	<0.0001^***^
SW AM2	0.214 (0.13–0.29)	<0.0001^***^
SW AM3	0.195 (0.11–0.27)	<0.0001^***^
SW AM4	0.243 (0.16–0.32)	<0.0001^***^

** *P < 0.005*,

*** *P < 0.0005*.

### Humanized Rat Model of Periodontitis and Treatment

**Figure 6:**
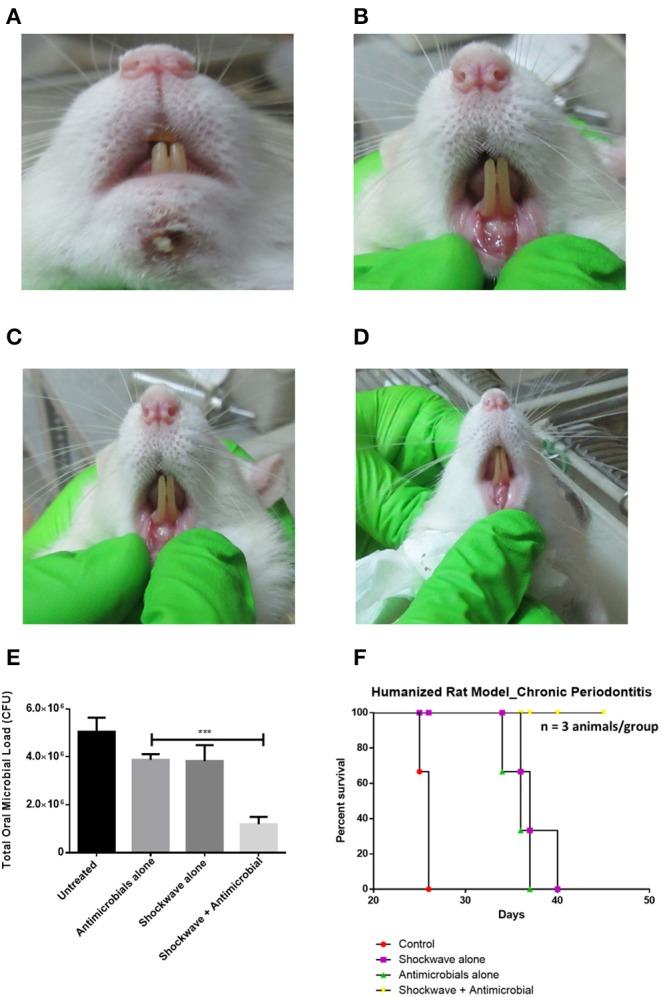
Evaluating the effect of shockwave treatment in a chronic periodontitis rat model. **(A–D)** Infected animals were exposed to a local shockwave at the rate of 1 shot per day for 7 days. They were treated either with shockwaves alone, antimicrobials alone or a combination of both. The control animal was left untreated. The shockwave and animicrobial alone treated animal showed disease progression whereas the animal treated with shockwaves followed by antimicrobial agent showed complete cure after the course of treatment. **(E)** The total CFU at the site of infection was also estimated. **(F)** An independent set of animals were tested for their survival post infection and treatment. Shockwave and antimicrobial treated animals survived whereas all the other treatment groups succummed to infection at different times. ***P < 0.0005.

Sprague-Dawley rats were injected with a mixed culture derived from chronic periodontitis patients in the sub-gingival region with a 26G syringe (Figures 5A,B). The animals were incubated under controlled conditions for the disease to establish. Twenty-one days post injections, the animals were examined for periodontitis like disease. Inflammation and pus formation were clinically evaluated in the animals at the sight of injection. The total bacterial load enumerated from the site of infection also indicated a successful disease establishment (Figure 5B). The animals were further subjected to either systemic antimicrobials described previously or were subjected to a combination of localized shockwaves and antimicrobial agents. The animals were treated for 10 days on a daily once basis. On day 14 it was observed that the animals treated with shockwaves and antimicrobial agents showed maximum recovery and the disease symptoms were found to be receding (Figures 6A–D). Whereas, the control group and the group treated with antimicrobial agents alone did not any a significant recovery of the disease. The total microbial load was enumerated from the site of infection post treatment. It was found that the total microbial load reduced significantly in the shockwave and antimicrobial agent treated group of animals as compared to others (Figure 6E). One cohort of animals was also evaluated for survival post treatment. The animals post infection were treated as described earlier. It was observed that the untreated animals as well as animals treated with antimicrobial agents alone succumbed to the infection by day 40 whereas, the animals treated with shockwaves in combination with antimicrobials survived even till day 45 and further (Figure 6F).

## Discussion

Periodontitis is an infectious disease caused by bacterial biofilm and not by planktonic pathogens. It is well-known that administration of local and systemic antibiotics does not reduce/eradicate the bacteria because the presence of biofilm creates a protective niche around them. It has been proven that the antiseptics are more effective on the disrupted biofilm performed either with manual or ultrasonic instruments (Anwar et al., 1992; Marsh, 2004). *In situ* oral biofilm models are the “gold standard” as they permit the generation and testing of biofilms under their native environments, reflecting clinical scenario. *In situ* oral biofilm models can be studied under two main categories, firstly those in which the biofilm develops directly on to the subject's tooth and next the models that rely on the development of biofilms on surfaces held within the mouth or on intra-oral splints. The main drawback of using *in vivo* biofilm model system being the need of volunteer and the biofilm formation (Ali et al., 2006). In addition, all the devices used in this system must test the volunteer's experience and the microbiological differences between the tooth-formed biofilm and the device-formed biofilm. This has led to development of various *in-vitro* biofilm model systems being, constant depth film fermenter (CDFF) (Atkinson and Fowler, 1974), artificial mouth model (Sissons et al., 1991), Microtitre multi-well plates, Calgary Biofilm Device (CBD) (Ceri et al., 1999; Ali et al., 2006), Sorbarod Biofilm Model System (Ledder et al., 2006). All these different biofilm models present with their own advantages and limitations. Very few subgingival biofilm models have been reported in the dental literature till date, probably due to the inherent difficulties in obtaining reproducible growth of these microorganisms and the growth medium being one of the key factors in *in vitro* biofilm development (Guggenheim et al., 2001). In this study, an attempt has been made to develop an *in vitro* biofilm model using six bacterial species simulating the composition of the *in vivo* subgingival plaque. *Actinomyces naeslundii, Streptococcus oralis, P. gingivalis, Veillonella parvula, Fusobacterium nucleatum*, and *Aggregatibacter actinomycetemcomitans*, are frequently found in the subgingival plaque (Smith et al., 1989; Ximénez-Fyvie et al., 2000; Ledder et al., 2006, 2007; Paster et al., 2006). These selected bacterial species also exemplify a wide range of physiological and metabolic characteristics, representing a diverse array of oral bacteria. With the use of Brain-heart infusion broth (BHI), which is a highly nutritious growth medium, for culturing fastidious and non-fastidious anaerobic microorganisms, together with the inoculated six selected bacterial species on natural saliva-coated HAP discs in anaerobic conditions, we were able to develop a biofilm which closely followed the design of (Sánchez et al., 2011). This study also provides evidence that the *in vitro* biofilms shares characteristics of *in vivo* biofilms. For example, the SEM and AFM images demonstrated dense complex biofilms over the HAP disks and the coverslips. Well-established 6 periodontopathogens were consistently recovered from the formed biofilms which was confirmed by PCR, further validating the model. The relation of increased antimicrobial resistances and reduced susceptibilities in biofilms is well-known. Very few investigations have documented the magnitude of the difference in antibiotic susceptibilities between biofilm and planktonically grown bacteria. Most of the documentation is exclusively been limited to mono-species biofilms of *Staphylococcus aureus* (Cerca et al., 2005; Jefferson et al., 2005; Nishimura et al., 2006) and *Pseudomonas aeruginosa* (Field et al., 2005; Hill et al., 2005; Abdi-Ali et al., 2006; Ali et al., 2006). These biofilms are inherently resistant to antibiotics either due to reduced penetration into the biofilm or because of secretion of certain enzymes like b-lactamases or binding of the antibiotic agent by the exopolysaccharide matrix (De Beer et al., 1994; Suci et al., 1994). The microenvironment within the biofilm differ in pH, metabolic activity and oxygen diffusion. The physical and structural disruption of biofilm is essential for the antimicrobials to have beneficial effect. In this study we used four clinically relevant antimicrobial agents namely amoxicillin-metronidazole, tetracycline, sodium hypochlorite and chlorhexidine. Extracorporeal shock wave therapy (ESWT) has been widely used in medical practice, for the management of cholelithiasis, urolithiasis, dermal wounds, various musculoskeletal conditions, and orthopedic conditions. The documented evidence on the use of ESWT in periodontics, however, is very limited. *In-vitro* studies have shown that shockwaves can be used as an adjunct in the regeneration of periodontal tissues following periodontal disease, at higher energy levels shockwaves exhibit bactericidal effect, and has a potential to remove calculus (Müller et al., 2011). A recent study has shown that the shockwaves are efficient in disrupting drug resistant biofilms both *in-vitro* as well as in-vivo (Gnanadhas et al., 2015). Here, in this study, we tested the role of shockwaves in disrupting biofilms formed on dental surfaces like a condition of periodontitis *in vivo*. It was observed by SEM and AFM analysis that on exposure to shockwaves the biofilm structure was disrupted, and complexity of biofilm was no longer maintained. SW alone (0.15 ± 0.31) had reduced the biofilm load when compared to Control biofilm (0.26 ± 0.15) but did not significantly affect the biofilms. The same was observed when the biofilms were treated with antimicrobials alone. Antimicrobials when used adjunctive to shockwave application showed a significant reduction in biofilm biomass irrespective of antimicrobial agent used. All the antimicrobial agents in the SW-AMA (Group IV) showed similar reduction of biofilm with highest efficacy showed by SW-CHX group (0.02 ± 0.003, *p* = 0.02) when compared to all other test groups. It is hypothesized that the exposure to the shockwaves ruptured the extracellular polymeric substance (EPS) surrounding the biofilm, liberating bacteria and possibly increasing access to the antimicrobial agents. Also, shockwaves themselves could have some bactericidal effect. Moving ahead, we established a humanized rat model of chronic periodontitis (Oz and Puleo, 2011). We were successful in establishing a human like periodontitis condition in rats which was clinically acceptable. We treated the rats with shockwaves, antimicrobials alone and a combination pf both. It was found that post treatment, the animals treated with shockwaves or antimicrobials alone did not show significant recovery from the disease. Whereas, the animals treated with a combination of shockwaves and antimicrobials showed maximum recovery from the disease. It was also seen in an independent experiment that the animals treated with a combination of antimicrobials and shockwaves did not succumb to chronic periodontitis as opposed to the untreated, shockwave alone and antimicrobial alone treated animals which succumbed to the disease. The results of this *in-vitro* and *in vivo* study form the platform to develop a novel method to manage chronic periodontitis at the clinical level soon.

## Data Availability Statement

The raw data supporting the conclusions of this manuscript will be made available by the authors, without undue reservation, to any qualified researcher.

## Ethics Statement

The animal study was reviewed and approved by Central Animal Facility, IISc.

## Author Contributions

AD and DC conceived the study. AD designed, performed experiments, and wrote the manuscript. CT collected patient samples and assisted in performing experiments and manuscript preparation. DC supervised the work. JG provided shockwave device. All the authors read and approved the manuscript.

### Conflict of Interest

The authors declare that the research was conducted in the absence of any commercial or financial relationships that could be construed as a potential conflict of interest.

## Abbreviations

SEM: Scanning Electron Microscopy
AFM: Atomic Force Microscopy
EPS: Extracellular Polymeric Substances
BHI: Brain Heart Infusion
AMX: Amoxycillin
MTZ: Metronidazole
TET: Tetracycline
CHX: Chlorhexidine.
